# Correlation between alterations in resting‐state functional alterations of brain and cerebrospinal fluid pathological markers in patients with Alzheimer's disease

**DOI:** 10.1002/alz.084078

**Published:** 2025-01-09

**Authors:** Hui Zhao, Cheng Bing Gong

**Affiliations:** ^1^ Department of Neurology, Affiliated Drum Tower Hospital, Nanjing University Medical School, Nanjing China; ^2^ The Affiliated Drum Tower Hospital of Nanjing University Medical School, Nanjing China; ^3^ Department of Neurology, Nanjing Drum Tower Hospital, The Affiliated Hospital of Nanjing University Medical School, Nanjing, Jiangsu China

## Abstract

**Background:**

The aim of this study was to explore the correlation between brain functional alterations and cerebrospinal fluid (CSF) pathological biomarkers in Alzheimer's disease (AD) patients.

**Method:**

A total of 39 individuals were recruited, including 23 AD patients and 16 control subjects. All subjects underwent a battery of neuropsychological examinations, CSF measurement and multimodal magnetic resonance imaging scans. Independent component analysis was used to investigate the variations of functional connectivity (FC) between the two groups by utilizing the resting‐sate functional MRI (RS‐fMRI) data. Differences in ALFF and ReHo between the two groups were also calculated. Then correlation analyses were used to estimate the possible association between functional alterations and CSF β‐amyloid (Aβ_1‐42_.) and tau.

**Result:**

Multiple inter‐ and intra‐network functional connections were altered in AD patients compared to the Non ADCI group. In the AD group, ALFF decreased in the right Superior Frontal Gyrus, Middle Frontal Gyrus, left superior temporal gyrus. and increased in the right cerebellum anterior lobe, and caudate nucleus as compared to Non‐AD CI group (p < 0.001). In addition, ReHo decreased in the right insula and left middle temporal gyrus (p < 0.001). Dynamic fluctuations of CSF Tau were observed to be associated with changes in FC between VN and PCC, FC of SMN, as well as the altered ReHo in the right insula and left middle temporal gyrus. Compared the AD group with the non‐AD CI group, the aforementioned altered functional brain connectivity, with the exception of FC in the PCC and VN, was significantly associated with a decrease in CSF Aβ_1‐42_.

**Conclusion:**

AD patients have exhibited functional changes in more than one brain region. These changes are linked to the variations of Tau protein and Aβ_1‐42_ in CSF, with Aβ_1‐42_ having a particularly large impact on brain network function. From an imaging standpoint, this study validated the impact of pathological changes on brain function in AD.

